# The impact of central corneal thickness on intraocular pressure among Ethiopian glaucoma patients: a cross-sectional study

**DOI:** 10.1186/1471-2415-12-58

**Published:** 2012-11-27

**Authors:** Yeshigeta Gelaw

**Affiliations:** 1 Department of Ophthalmology, College of Public Health and Medical Sciences, Jimma University, Jimma, Ethiopia

**Keywords:** Intraocular pressure, Central corneal thickness, Glaucoma, Pachymeter

## Abstract

**Background:**

Raised intraocular pressure (IOP) is the only causal risk factor for glaucoma that can be therapeutically manipulated to change the course of the disease process. Though Goldman applanation tonometry (GAT) is the “gold standard” for IOP measurement, readings of IOP with GAT are affected by central corneal thickness (CCT). The aim of this study is to determine the impact of CCT on IOP among Ethiopian glaucoma patients.

**Methods:**

It was a multicenter cross-sectional study and all glaucoma patients visiting their respective eye clinic during the study period were included. A total of 199 randomly selected glaucomatous eyes from 199 patients aged 18 years and above were employed. The CCT was measured by OcuScan™ RxP Ophthalmic Ultrasound and IOP was measured with Goldmann applanation tonometer. Linear regression and bivariate correlation analysis were carried out and level of significance was taken at 5%.

**Results:**

The mean IOP was 19.46(±7.05) mmHg and mean CCT was 508.07(±33.26) μm. The mean IOP for primary open angle glaucoma (POAG), ocular hypertension (OHT), normal tension glaucoma (NTG), pseudoexfoliative glaucoma (PXG) and primary chronic angle closure glaucoma (PCAG) patients was 19.22 mmHg, 21.39 mmHg, 14.33 mmHg, 33.25 mmHg and 14.75 mmHg respectively. The mean CCT values were 502.24 μm (POAG), 524.32 μm (OHT), 500.75 μm (NTG), 579.00 μm (PXG) and 530.25 μm (PCAG). Age of the patient and glaucoma surgery had an influence on corneal thickness. A positive relationship was found between CCT and IOP (p < 0.001).

**Conclusions:**

The mean CCT of Ethiopian glaucoma patients is thin in comparison to other ethnic groups and patients with OHT have thicker corneas than POAG patients. Hence determination of CCT for each patient is necessary in the up-to-date glaucoma management.

## Background

Glaucoma is the second leading cause of visual loss in the world [1] and the third leading cause of blindness in Ethiopia [2]. By the year 2020, it is predicted that the number of people with open angle glaucoma and angle closure glaucoma in Africa will be 8,359,451 and Africa will have the highest ratio (4.39%) of glaucoma to adult population [1]. Ethiopia, one of the highly populated African nations, with population of 74 million [3] will therefore have a large number of people with glaucoma.

Intraocular pressure (IOP) is a key element in the management of glaucoma [4] and it should, therefore, be measured using a reliable technique with high degree of accuracy. Though Goldman applanation tonometry (GAT) is the most widely used and current “gold standard” for IOP measurement [4], readings of IOP made with GAT are affected by central corneal thickness (CCT) [5]. Goldman and Schmidt felt that significant CCT variations occurred only rarely in the absence of corneal disease and thus assumed a "normal" CCT of 500 μm for their instrument [6]. However, studies have shown that there is variation in the mean CCT among individuals with healthy eyes [5,7], in patients with different types of glaucoma [8], and presence of pseudo-exfoliation syndrome [5]. Several studies have suggested 0.014–0.179 mmHg/0.01 mm increase in CCT. This small change could be clinically significant especially in individuals predisposed to glaucoma [9,10]. Moreover, failure to adjust IOP for CCT variation could lead to inappropriate targeting of IOP and setting targets very high for patients having thinner corneas and very low for those with thicker corneas.

Studies done in developed world have shown that people of African descent had increased risk of developing glaucoma, and primary open angle glaucoma is significantly more common, develops earlier, and is more severe in blacks than whites. However, CCT data among African in general and Ethiopian glaucoma patients in particular are scare or lacking. Hence this study endeavored to establish the CCT among Ethiopian Glaucoma patients with the aim of improving the management of glaucoma and it would also serve as a catalyst for ophthalmologists and eye-care workers in Ethiopia and the region in mainstreaming the clinical decision-making problem of glaucoma treatment in relation to CCT and IOP.

## Methods

A cross sectional hospital based study was conducted among Ethiopian glaucoma patients in three eye care settings (Jimma University Specialized Hospital, Menelik II Hospital and Zenebework-Alert Hospital) in Ethiopia from 26 May to 21 June 2007. All previously diagnosed glaucoma/glaucoma suspect patients who visited their respective eye clinic for follow up during the study period were included in the study. The eye to be examined in each patient was selected by lottery method. The diagnosis and classification of glaucoma patients were done based on IOP value, presence/absence of pseudoexfoliation, gonioscopy, optic disc evaluation and plus or minus visual field testing. A structured questionnaire was used to collect data from volunteering glaucoma patients with normal anterior segment aged 18 years and above. Patients not willing to participate, and those with major corneal pathology, previous intraocular (except trabeculectomy) and or corneal surgery were excluded from the study.

Five consecutive ultrasound pachymetry (OcuScan® RxP Ophthalmic Ultrasound System, Alcon Laboratories) measurements of CCT were obtained from the selected eye and a mean value was then computed and recorded in micrometers. All measurements were performed under topical anesthesia (amethocaine hydrochloride 2%). Then, two corresponding GAT measurements were obtained with the use of fluorescein staining and an average value was recorded in mmHg. The two IOP measurements were taken 15 minutes apart to rule out any tonometric effect on the pressure. The tonometer dial was set between 1 and 2 graduation marks of the prism and care was taken to avoid inaccurate readings from inappropriate fluorescein pattern resulting from excessive or insufficient fluorescein, and pressure on the globe by the examiner or patient squeezing the eyelids. The tonometer tip and the pachymeter probe tip were cleaned with dry cotton followed by swabbing the tips thoroughly with an alcohol prep pad and allowing it to dry for 10 minutes to reduce the risk of cross-infection.

Data were analyzed using SPSS for windows version 16.0. Linear Regression and correlation analyses were done as required. The CCT values in each age, sex, glaucoma type and ethnic groups were compared using t-test and One-Way ANOVA. P-value <0.05 was taken to be statistically significant.

This study was approved by the Research and Ethical Clearance Committee of Jimma University and carried out in compliance with the Helsinki Declaration (2006). Informed consent was obtained from each study participant and confidentiality of responses was assured and maintained.

## Results

A total of 199 randomly selected glaucomatous eyes from 199 consecutively recruited patients aged 18 years and above were included in the study. Among the study subjects, one hundred twenty three (61.8%) were males and 76(38.2%) were females. About sixty eight per cent were Semitic followed by Cushitic 49 (24.6%) (Table 1).

**Table 1:** Socio-demographic characteristics of the study subjects (N = 199)

**Variable**	**No**	**%**
**Age (years)**		
15-30	4	2.00
31-45	26	13.10
46-60	76	38.20
61-75	78	39.20
≥76	15	7.50
**Sex**		
Male	123	61.80
Female	76	38.20
**Ethnicity**		
Semitic	135	67.80
Cushitic	49	24.60
Omotic	7	3.50
Nilo-Saharan	1	0.50
Mixed	7	3.50
Total	199	100.00

The majority 148(74.4%) of patients had primary open angle glaucoma (POAG) followed by ocular hypertension (OHT) 31(15.6%) and normal tension glaucoma (NTG) 12(6%) as shown in Table 2. Sixty (30.2%) of the patients had trabeculectomy done and the rest 139(69.8%) did not have any surgery done. Eighty seven (43.7%) of the patients were on combined Timolol and Pilocarpine, 72(36.2%) on Timolol and 40(20.1%) of them were not on treatment. Regarding other health problems 6(3%) of the study subjects were diabetic, 8(4%) diabetic and hypertensive while the rest did not have any known medical illness.

**Table 2:** Type of glaucoma among the study subjects (N = 199)

**Glaucoma type**	**No**	**Percent**
POAG^†^	148	74.4
OHT^‡^	31	15.6
NTG^§^	12	6.0
PCAG^¶^	4	2.0
PXG^Φ^	4	2.0
Total	199	100.0

^†^*POAG: primary open angle glaucoma;*^‡^*OHT: ocular hypertension;*^§^*NTG: normal tension glaucoma;*^¶^*PCAG: Primary chronic angle closure glaucoma;*^Φ^*PXG: pseudoexfoliative glaucoma.*

**Figure 1:**
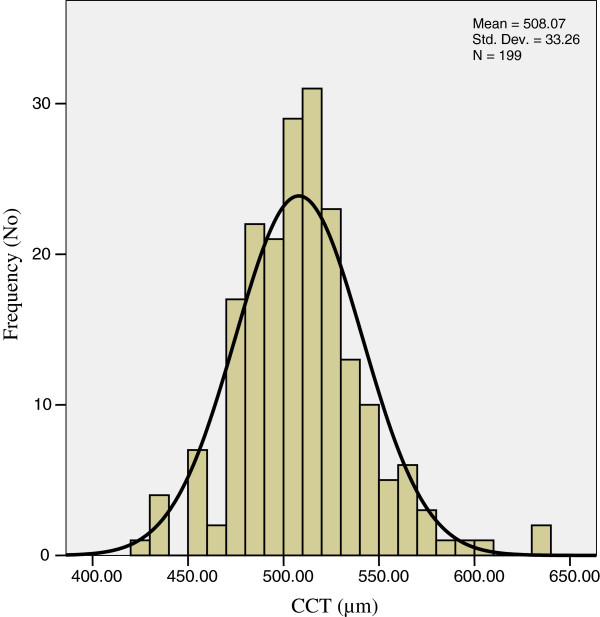
Distribution of CCT (μm) in the study subjects.

The mean age of the study population was 59.38 years (SD ± 12.15 years), mean IOP 19.46 mmHg (SD ± 7.05 mmHg), mean CCT 508.07 μm (SD ± 33.26 μm), and mean duration of glaucoma 38.77 months (SD ± 50.44 months). The distribution of CCT observed in the study subjects resembled a Gaussian curve with very slight departure from the mean, i.e. skewed towards the right (skewness =0.57) as shown in Figure 1.

The mean IOP in males was 19.72 mmHg (SD ± 6.98 mmHg) and in females 19.03 mmHg (SD ± 7.19 mmHg), and there was no statistically significant difference in mean IOPs (p = 0.506, 2-tailed, Independent Student T-test).

The mean CCT in females was 512.25 μm (SD ± 35.20 μm), which was slightly higher than the mean CCT in males 505.48 μm (SD ± 31.87 μm) but this difference in mean CCTs was not statistically significant (p = 0.164, 2-tailed, Independent Student T-test). The mean CCT in patients who had trabeculectomy was 524.27 μm and those who didn’t have any surgery was 491.87 μm (p < 0.05, 2-tailed, Independent Student T-test).

The mean IOP for POAG patients was 19.22 mmHg (SD ± 6.65 mmHg), and for OHT patients 21.39 mmHg (SD ± 7.99 mmHg), NTG patients 14.33 mmHg (SD ± 2.53 mmHg), pseudoexfoliative glaucoma (PXG) patients 33.25 mmHg (SD ± 2.50 mmHg) and primary chronic angle closure glaucoma (PCAG) patients 14.75 mmHg (SD ± 4.11 mmHg) (Table 3).

**Table 3:** Mean IOPs and CCTs versus type of glaucoma among the study subjects

**Type of glaucoma**	**Mean IOP(mmHg)**	**Mean CCT(μm)**
POAG^†^	19.22	502.24
OHT^‡^	21.39	524.32
NTG^§^	14.33	500.75
PCAG ^¶^	14.75	530.25
PXG^Φ^	33.25	579.00

^†^*POAG: primary open angle glaucoma;*^‡^*OHT: ocular hypertension;*^§^*NTG: normal tension glaucoma;*^¶^*PCAG: Primary chronic angle closure glaucoma;*^Φ^*PXG: pseudoexfoliative glaucoma.*

The mean CCT for POAG patients was 502.24 μm (SD ± 29.34 μm), and for OHT patients 524.32 μm (SD ± 36.81 μm), NTG patients 500.75 μm (SD ± 15.38 μm), PXG patients 579.00 μm (SD ± 63.51 μm) and PCAG patients 530.25 μm (SD ± 25.89 μm) (Table 3).

The mean CCT was slightly higher in the age group 15–30 years of age (522 ± 20.78 μm) and lower in the age group of 76 years and above (493.00 ± 35.84 μm) as shown in Table 4. However, this change in the mean CCTs was not statistically significant (p > 0.05, One-Way ANOVA).

**Table 4:** Central corneal thickness versus grouped-age distribution (N = 199)

**Age (years)**	**Central Corneal thickness (μm)**		
	**N (%)**	**Mean**	**SD**
15-30	4(2.00)	522.00	20.78
31-45	26(13.10)	504.54	27.33
46-60	76(38.20)	516.57	35.37
61-75	78(39.20)	503.14	31.27
≥76	15(7.50)	493.00	35.84

**Figure 2:**
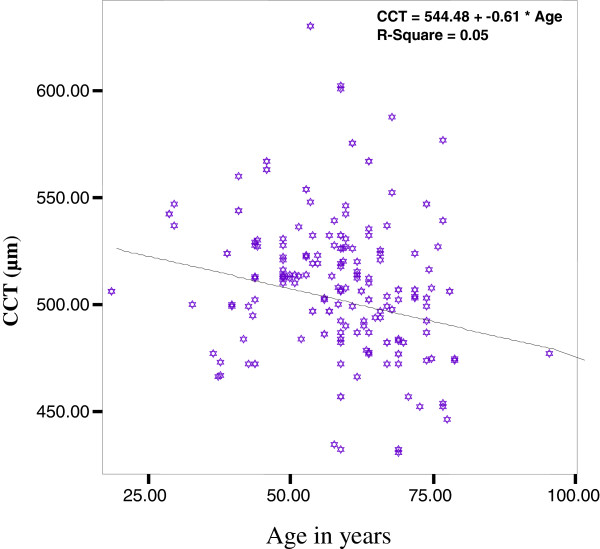
Relation between CCT (μm) and the age (years) in the study subjects.

There was a statistically significant change of CCT with age (r = 0.050, p = 0.001) and CCT showed a decline as age increases (Figure 2).

**Figure 3:**
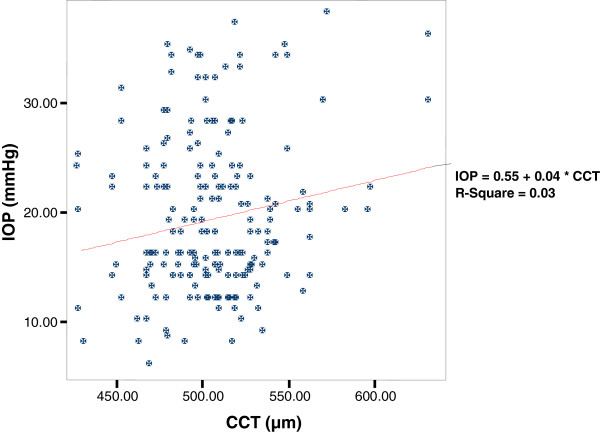
Relation between CCT (μm) and IOP (mmHg) in the study subjects.

This study revealed a statistically significant linear relationship between CCT and IOP as per the linear regression analysis(r = 0.031, p = 0.013). Furthermore, in a Bivariate correlation analysis, it was found that the CCT was correlated linearly with intraocular pressure values (ρ =0.175, p < 0.05) as shown in Figure 3.

Age of the patient (r = 0.050, p = 0.001) and glaucoma surgery (r = 0.031, p = 0.013) were also showed statistically significant relationship with CCT (Figure 2).

Ethnicity (r = 0.016, p = 0.076), sex (r = 0.010, p = 0.164), glaucoma type (r = 0.003, p = 0.465), duration of glaucoma (r = 0.000, p = 0.778), and presence of pseudo-exfoliation (r = 0.000, p = 0.884) showed no statistically significant relationship with CCT. Similarly, there was no statistically significant relationship between IOP and age, sex, ethnicity, and presence of pseudo-exfoliation within these study subjects (p > 0.05). However, type of glaucoma (r = 0.033, p = 0.010), and duration of glaucoma (r = 0.040, p = 0.005) showed statistically significant relationship with IOP.

## Discussion

Besides optic disc evaluation and visual field testing, the diagnosis of ocular hypertension, primary glaucoma and normal-tension glaucoma is based on an arbitrary IOP cutoff point of 21 mmHg [11]. This cutoff is made on the basis of statistical grounds, principally for screening purpose rather than as a diagnostic criterion. It is nevertheless in clinical use and any factor that changes the estimates of IOP can lead to a misclassification of the patient or predisposed individuals [12].

In this study it was found that the mean CCT and IOP among Ethiopian glaucoma patients were 508.07 μm and 19.46 mmHg respectively. This study also showed a statistically significant association between CCT and IOP. Furthermore, CCT was correlated linearly with IOP values (ρ =0.271, p < 0.001). In this study IOP was manipulated with surgery or medication in some, and not in others, and hence this result should be interpreted cautiously as correlation between baseline untreated IOPs and CCT is much more sensible. Moreover, there is a very large scatter of values; it is, therefore, not possible to apply a “linear wisdom” from this mathematical linear correlation.

Though the mean CCT of this study is significantly lower than the reported mean CCT of whites, it is in agreement with studies done among Africans and African-Americans [4,7,11,13-18] which reported thinner CCT. For example, studies done on healthy eyes of Ethiopians [17] and Sudanese [18] found a mean CCT of 518.68 ± 32.92 μm and 530.2 ± 58.1 μm respectively while the Ocular Hypertension Treatment Study (OHTS) [4] reported thinner corneas among African-American subjects with mean CCT of 555.7 ± 40.0 μm as compared to Caucasians with mean CCT of 573 ± 39 μm. This concurs with the findings of La Rosa F [7], Muir KW [15], Aghaian E [16].

OHTS also found that CCT is a risk factor for glaucoma and a strong predictor for progression from ocular hypertension to glaucoma [4]. Studies done in developed world have shown POAG is more severe in blacks than whites [19]. The thin CCT in this study population will, therefore, compound the risk of vision loss from glaucoma.

In this study, it was found that the mean CCT of PXG, OHT and PCAG patients is higher as compared to POAG and NTG patients and this is in accordance with previous studies [8,20-22] which reported that patients with OHT and PXG have thicker CCT than those with NTG and POAG. However, this comparison should not be over emphasized as the number of PXG and PACG eyes was small compared to other diagnosis groups, and this is the drawback of this study.

In this study, it was also found that patients who had trabeculectomy had thicker cornea than those who did not have any surgery. This might indicate that incisional surgeries or intraocular operations contribute to the higher readings possibly due to endothelial cell loss with subsequent altered hydration [23]. The endothelium does, however, have the capacity to remodel itself with a more stable configuration from three months post operative period and onwards. Knowing how old the surgery is, therefore, important to understand the matter. But this parameter was not entertained in this study. Since PXG and PACG patients tend to require trabeculectomy more often than other types of glaucoma, one could also attribute the thicker CCT findings among patients who had trabeculectomy to their initial thick CCT. This is, however, not the case in this study as patients with PXG and PACG accounted only 4% but those who had trabeculectomy were 30%. Another possibility is that even among the POAG patients those with higher CCT are expected to have initially presented with higher untreated IOP, and this higher IOP may have influenced the clinical decision making between medication and surgery. Comparison between CCT values before and after surgery could have been more informative, but none of the study patients had a prior record of CCT.

Though previous studies [24] done among whites suggest that there is no considerable change in CCT after infancy, this study demonstrated a statistically significant change of CCT with age and there was a decline of CCT as age increases. This finding is in agreement with studies [17,25,26] done among non-whites which showed a significant reduction of CCT with age.

## Conclusions

The mean CCT of Ethiopian glaucoma patients was found to be thinner as compared to Caucasians and the linear relationship between CCT and IOP was statistically significant. Patients with OHT and PXG had thicker corneas and higher IOPs than POAG and NTG patients. Age of the patient and glaucoma surgery had an influence on corneal thickness.

Determination of the CCT and categorization of corneas of glaucoma patients as thin, average, or thick are, thus, relevant since CCT has an influence on GAT values of IOP, which is the main parameter in the diagnosis, treatment and follow-up of glaucoma. A pachymeter is, thus, necessary in the up-to-date glaucoma management. In cases where pachymeter is not available, other factors such as optic disc, visual field, etc. should be considered and not only the IOP value itself.

## Competing interests

The author declares that there is no competing interest.

## Authors’ contributions

YG conceived the design of the study; collected, analyzed, and interpreted the data; wrote the manuscript and approved the final version for publication.

## Pre-publication history

The pre-publication history for this paper can be accessed here:

http://www.biomedcentral.com/1471-2415/12/58/prepub
